# Checklist-Based Identification of Adverse Drug Reactions in Emergency Department Patients

**DOI:** 10.3390/medicines12040025

**Published:** 2025-10-17

**Authors:** Benjamin J. Hellinger, Thilo Bertsche, Yvonne Remane, André Gries

**Affiliations:** 1 Emergency Department/Observation Unit Leipzig University Hospital, 04103 Leipzig, Germany; 2 Pharmacy, Leipzig University Medical Center and Medical Faculty, 04103 Leipzig, Germany; 3 Drug Safety Center, Leipzig University and Leipzig University Medical Center, 04103 Leipzig, Germany; 4 Clinical Pharmacy, Institute of Pharmacy, Medical Faculty, Leipzig University, 04103 Leipzig, Germany

**Keywords:** emergency department, prescribed medication, non-specific complaints, adverse drug reaction, checklist-supported diagnosis

## Abstract

**Background:** Patients presenting at the emergency department (ED) have a wide variety of complaints. In some of those patients a possible reason for their complaints might be an adverse drug reaction (ADR). An appropriate identification of ADR in this setting is required to optimize drug therapy and to prevent serious harm deriving from an overlooked ADR. **Methods:** This retrospective study assessed medical records of patients for ADR as a reason for the ED presentation in two assessments. In the first assessment, medical records were evaluated for potential ADR leading to ED presentation with a predefined checklist by an examiner not involved in initial patient treatment. In the second assessment the same medical records were assessed for ADR identified by the physician in the initial patient presentation. Discrepancies in identified ADR were compared. For descriptive data analysis and statistical evaluation, the McNemar test was performed. **Results:** From 35,333 patients admitted to the ED, full data were available from 34,747 patients for evaluation. In those patients, 2071 (6.0%) ADR were identified as being the reason for ED presentation by using the checklist. In 828 (2.4%) patients, emergency department physicians had documented an ADR in the medical records. By using the checklist, ADR identification could be improved significantly as compared to routine care, at 6.0% vs. 2.4%, respectively (*p* < 0.001). The most common chief complaint in patients with an ADR was worsened general condition. Most common drug class causing ADR were antithrombotics. **Conclusions**: ADR seem to be overlooked in routine care since a significantly higher number of ADR were found by using a checklist-based method as compared to ADR documented as part of routine examination. Therefore, implementing the checklist in the routine process might improve ADR identification.

## 1. Introduction

Adverse drug reactions (ADRs) pose a significant challenge in today’s healthcare systems, contributing substantially to patient morbidity and increased healthcare costs [1,2,3,4]. ADRs, defined as harmful or unintended effects of a medication, are responsible for a notable proportion of hospital admissions and emergency department (ED) visits. It is estimated that ADRs account for approximately 5% of hospitalizations in developed countries [5]. Up to approximately half of ADRs may be preventable, depending on setting and preventability definitions [6]. In some cases, they may result in costly interventions such as prolonged hospital stays, additional treatments, or even the need for intensive care [7,8,9,10]. In a recent meta-analysis, patients with suspected ADRs had increased odds of all-cause mortality (OR 1.50, 95% CI 1.21–1.86) [11]. Furthermore, those patients had a significant difference in length of hospital stay by 4 days [11]. Despite these alarming figures, only 5–10% of ADR get reported to pharmacovigilance systems [12]. Some of them are entirely overlooked, thereby posing a significant risk to patient safety and complicating the management of chronic conditions [13,14]. In particular, the large proportion of older patients with multiple health conditions and resulting polypharmacy are at risk of experiencing negative outcomes from ADR [15,16,17].

One of the primary challenges in recognizing ADRs is the wide range of symptoms that they present, which can easily be mistaken for natural disease progression or the onset of new medical conditions [18,19]. For example, adverse effects such as fatigue, dizziness, or gastrointestinal issues are often attributed to underlying health problems rather than the medications themselves [18]. In EDs, where physicians must prioritize rapid diagnosis and treatment, the subtlety of some ADRs may result in them being overlooked or misdiagnosed. This risk is particularly prevalent in patients who take multiple medications simultaneously, due to the complex interactions that can arise from the combination of various drugs [17]. Additionally, the fast-paced environment of the ED may lead to incomplete or inaccurate medication histories, further complicating the identification of ADRs [20,21].

It has been frequently observed that standardized tools for healthcare providers to support the identification of ADRs are lacking. Consequently, they rely on clinical judgment, which is known to vary considerably among practitioners. The timely and appropriate identification of potential ADRs in the ED is of paramount importance for effective management, however. One potential solution to this problem would be to implement systematic monitoring tools designed to facilitate the identification of ADRs, particularly in patients at high risk [22]. Such tools could standardize the way healthcare professionals assess potential ADRs, ensuring that key signs are not missed during patient evaluations [23]. A structured checklist could assist clinicians in asking targeted questions regarding recent medication changes, monitoring drug interactions more effectively, and ultimately reducing the number of overlooked ADRs. By addressing the issue of missed ADRs through such strategies, healthcare systems could not only improve patient outcomes but also alleviate some of the financial and logistical burdens associated with ED visits and hospitalizations as a result of ADRs.

The aim of this study was to ascertain whether the implementation of a checklist would improve ADR identification in ED patients when compared to the results of routine care. Another objective was to determine in which ED visits ADR are overlooked and what drugs are the cause of these visits.

## 2. Materials and Methods

### 2.1. Study Setting and Patients

This retrospective study analyzed the medical records of adult patients aged 18 years and above who presented consecutively to the ED of a university hospital that offers tertiary care. Patients who presented to the ED between 1 January and 31 December 2019, and for whom a medical record was available were included. The medical record contained patient information on demographic parameters, chief complaint, laboratory values, pre-existing conditions, complaints at the ED visit, patient’s medication, results of diagnostic procedures, and discharge notes. Medical records from another hospital for secondary-transfer patients were excluded.

### 2.2. Study Protocol

**Figure 1:**
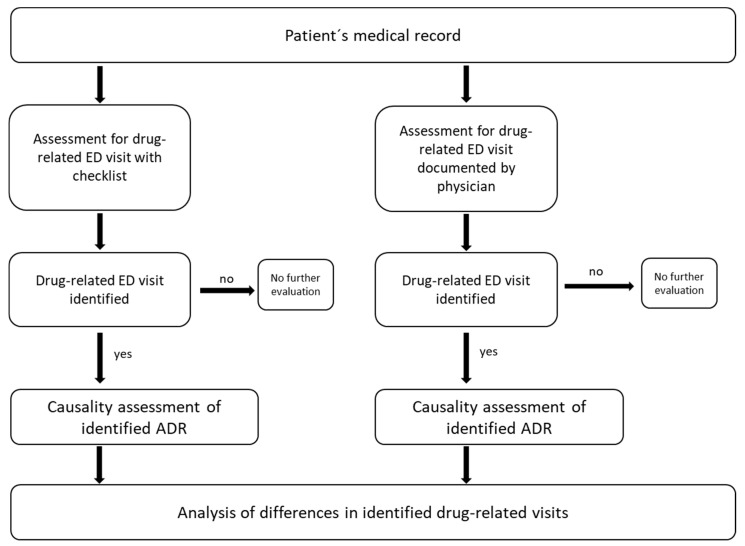
Workflow of retrospective evaluation.

This was a retrospective study based on two assessments. As the initial assessment, medical records were examined using a predefined checklist to determine whether any ADR was the possible reason for the patient presenting to the ED (checklist-based assessment: CL-A). In the second assessment, the same medical records were evaluated by the examiners to check whether the ED physician treating the patient in the ED had documented an ADR as the cause for admission (physician’s documentation-based assessment: PD-A) [Figure 1]. Each record was assessed twice, first in the CL-A and then in the PD-A. By conducting both assessments to the same records within the same uninterrupted timeframe, we eliminated any calendar-based separation between the assessments, thereby minimizing potential bias introduced by seasonal variation, changes in ED workflow, or staffing fluctuations.

In this study an ADR was defined as a noxious and unintended response to a medicine, in accordance with the definition of the European Medicines Agency [24]. Patients who presented to the ED due to an ADR caused by medication were considered as drug-related ED visits.

### 2.3. Assessment for Drug-Related ED Visit with Checklist (CL-A)

A multidisciplinary expert panel, consisting of ED physicians and clinical pharmacists with practical knowledge of ADRs, collaborated with pharmacists from the Drug Safety Center Leipzig to create a checklist. The checklist aims to establish a standardized clinical workflow for identifying drug-related ED visits. Literature regarding ADR risk factors, ADR identification, and already existing tools were reviewed and discussed in the expert panel [13,19,22,23,24,25,26]. Furthermore, recent ADR cases and clinical experience of the panel members were incorporated in the creation process. Points of consensus that should be considered for identifying ADR were implemented in the checklist [Table 1]. The checklist includes basic aspects such as evaluating the patient’s current medication but also takes ADR risk factors such as potentially inadequate medication into account. For specific issues, e.g., drug-drug interactions, sources of information that are used as a standard in the clinic were referenced in the checklist. As the final step of the checklist, the ADR was classified as either possible, probable, or certain for drug-related ED visits or unlikely for non-drug-related ED visits. This classification was based on the definitions provided by the WHO Uppsala Monitoring Centre and conducted by the expert panel [27]. After the checklist was finalized by the expert panel, no further adjustments were made during the retrospective medical record review, ensuring consistent application throughout the study. No pilot testing or formal validation procedures outside the expert panel were performed prior to its use in this study. Instead, the present retrospective review represents the first application of the checklist to a clinical dataset and serves as an initial evaluation of its feasibility and applicability.

An examiner not involved in patient treatment processed the checklist using the patient’s medical record. This process was conducted by a clinical pharmacist. The medical record included patient’s complaints at ED visit, laboratory values, medication, pre-existing conditions, and the results of diagnostic procedures. To preserve the neutrality of the examiner, discharge notes, including physicians’ diagnoses and subsequent recommendations were not provided in this assessment.

### 2.4. Assessment for Drug-Related ED Visit Documented by Physicians (PD-A)

In the second assessment, the examiner assessed the same medical records of patients for drug-related ED visits documented by the ED physician at the time in the discharge notes. A documented drug-related ED visit was defined either as an ADR given as diagnosis, a definitive description of an ADR in the discharge notes, or as a recommendation to discontinue or reduce the dose of a suspected drug, respectively. The expert panel conducted a WHO causality assessment for documented drug-related ED visits.

### 2.5. Data Analysis

Age, sex, need for hospitalization, and chief complaint of all ED patients were derived from the medical records, by the examiner. For identified drug-related ED visits, the number of drugs prescribed and drug class possibly responsible for ADR were also documented. Demographic data are shown as median with corresponding interquartile ranges (IQR). Drug-related ED visits identified in the two assessments were compared. Only drug-related ED visits that were classified as possible/probable/certain in the causality assessment were considered for analysis. McNemar test and relative risk calculation were performed using IBM SPSS 25. *p* < 0.05 was considered statistically significant.

## 3. Results

In the investigated time period, 35,333 patients were treated in the ED, and 34,747 medical records were available for further evaluation.

### 3.1. Patient Characteristics

The median age of the overall ED population (patients with ADR and patients without ADR) was 50 ([IQR] 31–71) years [Table 2]. CL-A patients had a median age of 72 ([IQR] 56–81.25) years and were taking seven ([IQR] 4–10) different drugs. Patients in whom a drug-related ED visit was identified in PD-A had a median age of 69 ([IQR] 49–81) years and they were taking six ([IQR] 3–9) drugs.

### 3.2. Adverse Drug Reactions (ADR)

Among the CL-A patients, 2071 ADR were identified as possibly being responsible for the patient’s ED admission. That would account for 6.0% of total ED visits. An ADR as the origin of the patient’s visit was documented in the medical record by the ED physician in 828 patients in PD-A, accounting for 2.4% of total ED visits. This difference was statistically significant in the McNemar test [OR = 83.9 (95% CI 50.3–139.7), *p* < 0.001, Cohen’s g = 0.49, large effect]. Assessing medical records with the checklist increased ADR detection by 3.6 percentage points, meaning that for every 28 patient records reviewed using the checklist, one additional ADR would be identified, compared to routine care (95% CI 26.5–29.6).

Furthermore, 1258 ADR were identified exclusively in CL-A, 15 ADR exclusively in PD-A, and 813 ADR were identified in both assessments. According to the causality assessment most ADR were classified as possible, followed by probable, and certain in CL-A as well as in PD-A [Table 2].

### 3.3. Chief Complaints

**Figure 2:**
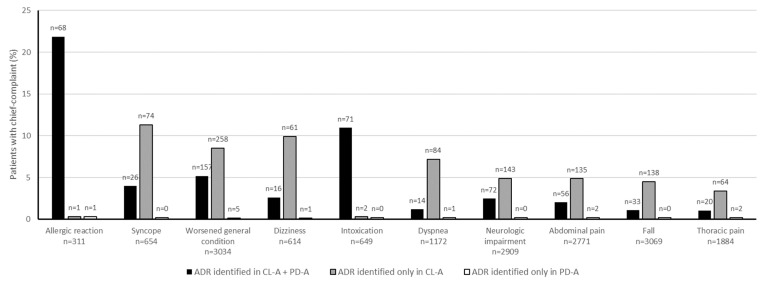
Top 10 chief complaints in whom an adverse drug reaction (ADR) was identified as cause of the emergency department (ED) presentation in the checklist-based assessment (CL-A) and physician documentation-based assessment (PD-A); ED patients in whom no ADR was identified are not displayed in the figure but implied in the total number of chief complaints.

The most common chief complaints in patients with drug-related ED visits are displayed in Figure 2.

### 3.4. Drugs

**Figure 3:**
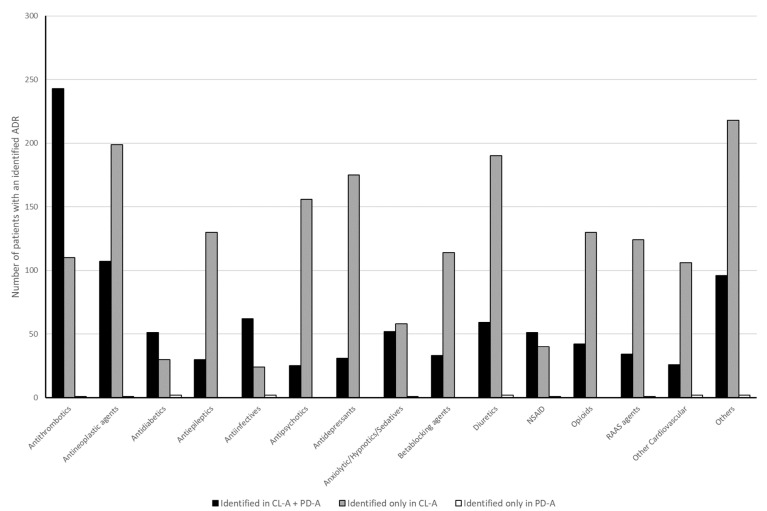
Drugs that were identified to have caused an adverse reaction (ADR) leading to emergency department visit in checklist-based assessment (CL-A) and physician documentation-based assessment (PD-A). NSAID, nonsteroidal anti-inflammatory drugs.

A total of 2761 drugs were identified as possibly being responsible for the ADR in CL-A. Most commonly identified drug classes were antithrombotic agents (13.3%), antineoplastic and immunomodulating agents (11.4%), and diuretics (8.2%) [Figure 3]. In PD-A, 957 drugs were identified to have caused the ADR. Antithrombotics (25.9%), antineoplastic and immunomodulating agents (11.6%), and anti-infective (6.7%) were implicated most often [Figure 3].

## 4. Discussion

Medications can cause ADR, potentially prompting patients to utilize the healthcare system, including the ED. When these individuals present at the ED due to an ADR, it is possible that neither the patient nor ED personnel initially recognizes the complaints as being drug related, therefore causing further patient harm and increased burden for the healthcare system [16,28]. Therefore, better strategies for managing ADR in ED patients are required. We investigated the frequency of ADRs documented in routine examinations and created a checklist for systematically improving the identification of ADR in ED patients. By using our checklist-based approach, the number of ADR identified increased by about 2.5-fold and was comparable to the prevalence of ADR in the ED found in previous studies [5].

### 4.1. ADR Identification

This study showed that ADRs represent a prevalent cause of ED presentation and that the implementing a standardized checklist might facilitate the identification of ADRs within the ED setting. Our findings indicate that 60.7% of patients had an ADR identified by the checklist that was not documented in the medical records by ED personnel. This suggests that such cases may have been overlooked in routine care, which is in line with findings from studies investigating ADR as the reason for hospitalization [29].

Keeping the median age of patients with ADR in mind, ADR mainly constitute a problem in older individuals who are taking multiple medications, which is in line with previous studies [16]. As these patients have multiple health conditions, a new symptom caused by a drug may not be recognized as an ADR but rather as a symptom of an underlying disease or even a new disease. It is therefore crucial to identify the ADR as soon as possible in order to prevent it from being treated as a new symptom of a disease with a new drug. Once the ADR has been identified, solutions for resolving it must be sought, such as reducing the dose, stopping the drug treatment, or switching the drug.

### 4.2. Chief Complaints and Identified Drugs

The most frequently reported chief complaint among patients with a drug-related ED visit is worsened general condition followed by neurologic impairment in both assessments. Particularly in patients with nonspecific symptoms such as fatigue or generalized weakness, an ADR might be difficult to identify. While antithrombotic agents represent the most frequently identified type of drug, the chief complaint documented, hematemesis/melena, is exceedingly rare. This illustrates that ADRs may not always manifest as overtly specific symptoms. Instead, they may present as general weakness or fatigue due to anemia.

The findings of this study indicate that drugs targeting the central nervous system (CNS), such as antidepressants or antipsychotics, were identified as causing ADR at a considerably lower frequency during routine examination when compared to the checklist-guided assessment. The existence of this discrepancy can be attributed to several factors. Firstly, these drugs may be responsible for nonspecific symptoms such as generalized weakness, dizziness or altered mental state by depressing the CNS, especially when multiple drugs are prescribed concurrently. The presence of these symptoms may result in a patient’s cognitive impairment, hindering their capacity to provide a comprehensive medical history, thereby impeding the identification of ADRs. Secondly, in the acute setting, emergency physicians potentially prioritize ruling out life-threatening causes, such as stroke, over considering potential pharmacologic effects. Finally, the discrepancy could also be attributed to the fact that drugs within this heterogenous group often possess a broad range of potential ADR, with which not every emergency physician is necessarily familiar [17,18]. The checklist guided assessment appears to mitigate both vague or nonspecific symptoms and potential knowledge gaps, leading to more consistent detection of CNS-related ADRs.

Diuretics being in 3rd place of the drugs responsible for ADR emphasizes that this class of drug should be prescribed and reviewed carefully, especially in older patients. Diuretics frequently cause hypovolemia and electrolyte disturbances, which are associated with high mortality in older patients [30]. Diuretics can also contribute to volume depletion, acute renal failure, and syncope by causing orthostatic hypotension. That diuretics and agents acting at the central nervous system are responsible for drug-related ED visits was shown previously in Switzerland in a population presenting with nonspecific complaints, and demands for a screening tool for these patients were issued [12].

In patients who have experienced a fall, an ADR as reason for the fall was potentially overlooked in 80% in routine care. Drugs should be reviewed critically to identify those that increase the risk of falls because they represent one of the best modifiable risk factors [31,32]. This applies to most of the drugs that influence the central nervous system, as they often cause dizziness, orthostatic hypotension, or extrapyramidal side effects [19]. Ways to improve awareness for drugs that increase the fall risk are needed in the ED setting to prevent recurrent visits.

There was a strong overlap between the chief complaints of intoxication and allergic reaction, as identified on the checklist, and those documented during the routine examination. This is because these complaints are highly specific, with few differential diagnoses. Consequently, if a drug is responsible for the complaint, it can be identified with reasonable certainty without the need for specific checklists. Furthermore, for drug classes such as antibiotics, antidiabetics, and antithrombotic agents, the overlap was strong in both assessments, indicating that physicians may be more familiar with the ADR for these agents.

While the present study did not directly assess the clinical severity of individual ADRs, it compared hospitalization rates of patients with an identified ADR in CL-A and PD-A. The observed percentage of patients who did get hospitalized subsequent to the ED visit was similar in CL-A and PD-A. This suggests that the checklist is capable of detecting clinically relevant ADRs, as opposed to minor events that are unlikely to require inpatient care.

These findings suggest that the assessment of the checklist can help identify clinically relevant ADR of drugs who emergency physicians might not be familiar with and especially in patients with non-specific symptoms. Unlike existing pharmacovigilance tools or scoring systems, which are primarily for reporting after an ADR has been identified, the checklist functions as a structured identification aid. It guides the examiner to systematically assess whether the patients’ symptoms might be drug-related, based on patient risk factors and drug risk factors. By taking these risk factors into account early in the diagnostic process, physicians may be more likely to consider ADRs as a possible cause, even before a definitive diagnosis is made. In this way, the checklist serves to complement the physicians’ routine care for those ADRs that are more prone to being overlooked in the fast-paced ED.

### 4.3. Causality

The majority of ADR in CL-A and PD-A was classified as “possible” in the WHO causality assessment. This finding emphasizes that it is often challenging to distinguish between a definitive ADR and underlying diseases causing a patient’s symptoms, especially in the ED where information on the subsequent course of the ADR is lacking and in older, multimorbid patients. While this uncertainty is inevitable to retrospective evaluation, it also gives rise to ethical considerations, as potentially clinically significant ADRs identified in CL-A were not reported to pharmacovigilance authorities in accordance with ethics approval. In a prospective setting, the utilization of the checklist should be accompanied by clearly defined processes for timely ADR reporting and clinical follow-up to ensure patient safety.

### 4.4. Outlook

The results of this retrospective evaluation suggest that implementing the checklist into the clinical practice of the ED could substantially reduce overlooked ADR. However, conducting the complete checklist is time consuming and might be difficult to assess by physicians alongside their routine care. Therefore, we propose different strategies to make the implementation feasible. First, to only conduct the checklist for high-risk patients, such as older adults, patients with general worsened condition or patients with polypharmacy, whose ADR seem to be overlooked the most often. Secondly, specialized trained personnel could be integrated in the ED team, such as trained nurses, clinical pharmacists or pharmacy technicians, whose primary task is assessing the checklist. Thirdly, with emerging digitalization, coupling the checklist with electronic health records and medication safety tools might be an approach to ensure checklist processing while keeping individual time expense low. For example, configuring warning alerts for patients with electrolyte imbalance in the laboratory values and diuretic use in their medication or flagging drugs with a fall risk in older patients who present after a fall. Together, these different approaches would make the implementation of the checklist in the fast-paced ED feasible and warrant prospective evaluation.

### 4.5. Summary

Compared to other studies investigating ADR in the ED, we found a similar prevalence of ADR at 6.0% by assessing medical records with a predefined checklist [5,9]. In these studies, clinical decision rules for ADR risk stratification and trained study personnel were used to identify ADR in ED patients. Therefore, a checklist-based identification might represent a new approach suitable for detecting ADR in ED patients. An extended medication assessment is necessary to accurately identify ADR. This process and evaluation using the checklist are time consuming and might not be feasible for ED personnel in a routine setting. The implementation of the checklist, in conjunction with suitably qualified and trained personnel to evaluate drug-related ED visits, therefore, appears to be desirable for the future.

### 4.6. Limitations

Despite the strong findings of this study, there are several limitations. First, the retrospective design of the study is such that the checklist is limited to the medication history documented in the medical record, leading to documentation bias. Consequently, ADRs resulting from drugs taken by the patient but not documented or asked for by the physician may have been overlooked in both assessments, ultimately leading to underreporting. This might especially be relevant for OTC-drugs and supplements, which are often only reported by the patient if asked directly. Secondly, it should be noted that ADRs identified by the physician when the patient presented at the ED but not documented in the medical record could not be recorded here. Consequently, the number of ADRs in routine care may be underrepresented. Thirdly, the retrospective assessment in CL-A and PD-A was conducted with the goal to identify drug-related ED visits, which implicates a risk of confirmation bias. However, the risk of bias is minimal due to the utilization of a standardized checklist, which was developed through an interdisciplinary and independent process, and a causality assessment conducted by a multiprofessional expert panel. Moreover, the consistency of the findings with those of previous studies lends support to the practical utility of the checklist. Finally, in the case of some outpatients, particularly trauma patients, no medication history was available, and information regarding the timeliness of the medication was lacking. This may have resulted in an underreporting of ADRs in this study.

## 5. Conclusions

This study shows that by using a checklist-based system, detecting ADR as a reason for a patient to visit the ED can be improved. Especially in older patients presenting to the ED who are on multiple drugs and in worsened general condition, ADR should be considered. In addition to high-risk medications such as chemotherapeutics and antithrombotic agents, drugs influencing the central nervous or the cardiovascular system should also be reviewed carefully. It is important to distinguish between an ADR and a new symptom of a disease in order to prevent polypharmacy and patient harm. Further investigations are needed to study the feasibility of implementing this checklist into routine processes in the ED.

## Figures and Tables

**Table 1:** Checklist for ADR identification derived from the expert panel.

Step	Examination	Tool
**#1**	Medical history	- Chief complaint - Anamnesis - Laboratory findings - Imaging processes
**#2**	Medication history (e.g., antithrombotics, diuretics, opioids, NSAIDs)	- Medication plan - OTC medication - Timeliness of present medication - Number of drugs prescribed - Medication changes in last 3 months
**#3**	Relation of medication ↔ symptoms (e.g., bleeding, falls, syncope, skin rash)	- Medication review - ADR, interactions (*source: UpToDate, ABDATA, SmPC*) - Potentially inadequate medication (*source: PRISCUS*)
**#4**	Relation of medication ↔ laboratory finding (e.g., electrolytes, renal function, transaminases)	- Medication review - Dosing adequate to renal function (*source: dosing.de*)
**#5**	Relation of medication ↔ other pathologic findings (electrocardiogram, imaging)	- Medication review
**#6**	Causality assessment (WHO UMC)	- Possible/Probable/Certain - Unlikely

**Table 2:** Patient characteristics of the overall emergency department (ED) population and patients with a drug-related ED visit.

	Overall ED Population (*n* = 34,747)	CL-A	PD-A
**Identified drug-related ED visits *n* (%)**		2071 (6.0)	828 (2.4)
**Age-in-years median (IQR)**	50 (31–71)	72 (56–81.25)	69 (49–81)
**Gender% (*n*)**			
Female	40.0 (14,053)	50.7 (1050)	51.1 (423)
Male	60.0 (20.694)	49.3 (1021)	48.9 (405)
**Non-trauma%**	48.1	85.3	88.9
**Hospitalization rate% (*n*)**	36.5 (12,683)	61.1 (1257)	62.3 (516)
**Top 10 chief complaints% (*n*)**			
Worsened general condition	8.7 (3032)	20.0 (415)	19.4 (161)
Neurologic impairment	8.4 (2909)	10.4 (215)	8.7 (72)
Fall	8.8 (3069)	8.3 (171)	4.0 (33)
Abdominal pain	8.0 (2771)	6.9 (143)	7.0 (58)
Syncope	1.9 (654)	4.8 (100)	3.1 (26)
Dyspnea	3.4 (1172)	4.8 (98)	1.8 (15)
Thoracic pain	5.4 (1884)	4.1 (84)	2.7 (22)
Intoxication	1.9 (649)	3.5 (73)	8.6 (71)
Allergic reaction	0.9 (311)	3.3 (69)	8.3 (69)
Dizziness	1.8 (614)	3.2 (67)	2.1 (17)
**WHO causality assessment% (*n*)**			
Possible		87.1 (1804)	69.8 (578)
Probable		8.4 (174)	19.0 (157)
Certain		4.5 (93)	11.2 (93)

## Data Availability

The datasets used and analyzed during the current study are available from the corresponding author on reasonable request.

## Abbreviations

The following abbreviations are used in this manuscript:

ADR	Adverse drug reaction
CL-A	Assessment of the medical record with checklist
ED	Emergency Department
IQR	Interquartile range
NSAID	non-steroidal anti-inflammatory drug
OTC	over the counter
PD-A	Assessment of physician’s diagnosis in medical record
SmPC	Summary of product characteristics
UMC	Uppsala Monitoring Centre
WHO	World Health Organization

## References

[1] Fasipe O.J., Akhideno P.E., Owhin O.S. (2019). The observed effect of adverse drug reactions on the length of hospital stay among medical inpatients in a Nigerian University Teaching Hospital. Toxicol. Res. Appl..

[2] Geller A.I., Lovegrove M.C., Shehab N., Hicks L.A., Sapiano M.R.P., Budnitz D.S. (2018). National Estimates of Emergency Department Visits for Antibiotic Adverse Events Among Adults-United States, 2011-2015. J. Gen. Intern. Med..

[3] Johansen A., Dickens J., Jones M., Richmond P., Evans R. (2011). Emergency department presentation following falls: Development of a routine falls surveillance system. Emerg. Med. J..

[4] Sultana J., Cutroneo P., Trifirò G. (2013). Clinical and economic burden of adverse drug reactions. J. Pharmacol. Pharmacother..

[5] Schurig A.M., Böhme M., Just K.S., Scholl C., Dormann H., Plank-Kiegele B., Seufferlein T., Gräff I., Schwab M., Stingl J.C. (2018). Adverse Drug Reactions (ADR) and Emergencies. Dtsch. Arztebl. Int..

[6] Hakkarainen K.M., Hedna K., Petzold M., Hägg S. (2012). Percentage of patients with preventable adverse drug reactions and preventability of adverse drug reactions--a meta-analysis. PLoS ONE.

[7] Patel T.K., Patel P.B., Bhalla H.L., Dwivedi P., Bajpai V., Kishore S. (2023). Impact of suspected adverse drug reactions on mortality and length of hospital stay in the hospitalised patients: A meta-analysis. Eur. J. Clin. Pharmacol..

[8] Komagamine J., Kobayashi M. (2019). Prevalence of hospitalisation caused by adverse drug reactions at an internal medicine ward of a single centre in Japan: A cross-sectional study. BMJ Open.

[9] Meier F., Maas R., Sonst A., Patapovas A., Müller F., Plank-Kiegele B., Pfistermeister B., Schöffski O., Bürkle T., Dormann H. (2015). Adverse drug events in patients admitted to an emergency department: An analysis of direct costs. Pharmacoepidemiol. Drug Saf..

[10] Pirmohamed M., James S., Meakin S., Green C., Scott A.K., Walley T.J., Farrar K., Park B.K., Breckenridge A.M. (2004). Adverse drug reactions as cause of admission to hospital: Prospective analysis of 18 820 patients. BMJ Open.

[11] Patel T.K., Patel P.B. (2018). Mortality among patients due to adverse drug reactions that lead to hospitalization: A meta-analysis. Eur. J. Clin. Pharmacol..

[12] Hazell L., Shakir S.A. (2006). Under-reporting of adverse drug reactions: A systematic review. Drug Saf..

[13] Nickel C.H., Ruedinger J.M., Messmer A.S., Maile S., Peng A., Bodmer M., Kressig R.W., Kraehenbuehl S., Bingisser R. (2013). Drug—Related emergency department visits by elderly patients presenting with non-specific complaints. Scand. J. Trauma Resusc. Emerg. Med..

[14] Dormann H., Criegee-Rieck M., Neubert A., Egger T., Geise A., Krebs S., Schneider T.H., Levy M., Hahn E.G., Brune K. (2003). Lack of awareness of community-acquired adverse drug reactions upon hospital admission: Dimensions and consequences of a dilemma. Drug Saf..

[15] Field T.S., Gurwitz J.H., Avorn J., McCormick D., Jain S., Eckler M., Benser M., Bates D.W. (2001). Risk factors for adverse drug events among nursing home residents. Arch. Intern. Med..

[16] Evans R.S., Lloyd J.F., Stoddard G.J., Nebeker J.R., Samore M.H. (2005). Risk factors for adverse drug events: A 10-year analysis. Ann. Pharmacother..

[17] Rawle M.J., Cooper R., Kuh D., Richards M. (2018). Associations Between Polypharmacy and Cognitive and Physical Capability: A British Birth Cohort Study. J. Am. Geriatr. Soc..

[18] Hohl C.M., Zed P.J., Brubacher J.R., Abu-Laban R.B., Loewen P.S., Purssell R.A. (2010). Do emergency physicians attribute drug-related emergency department visits to medication-related problems?. Ann. Emerg. Med..

[19] Hohl C.M., Robitaille C., Lord V., Dankoff J., Colacone A., Pham L., Bérard A., BPharm J.P., Afilalo M. (2005). Emergency physician recognition of adverse drug-related events in elder patients presenting to an emergency department. Acad. Emerg. Med..

[20] de Winter S., Spriet I., Indevuyst C., Vanbrabant P., Desruelles D., Sabbe M., Gillet J.B., Wilmer A., Willems L. (2010). Pharmacist- versus physician-acquired medication history: A prospective study at the emergency department. Qual. Saf. Health Care.

[21] Fitzgerald R.J. (2009). Medication errors: The importance of an accurate drug history. Br. J. Clin. Pharmacol..

[22] Hohl C.M., Badke K., Zhao A., Wickham M.E., Woo S.A., Sivilotti M.L.A., Perry J.J. (2018). Prospective Validation of Clinical Criteria to Identify Emergency Department Patients at High Risk for Adverse Drug Events. Acad. Emerg. Med..

[23] Haerdtlein A., Boehmer A.M., Karsten Dafonte K., Rottenkolber M., Jaehde U., Dreischulte T. (2022). Prioritisation of Adverse Drug Events Leading to Hospital Admission and Occurring during Hospitalisation: A RAND Survey. J. Clin. Med..

[24] European Medicines Agency Clinical Safety Data Management: Definitions and Standards for Expedited Reporting. https://www.ema.europa.eu/en/documents/scientific-guideline/international-conference-harmonisation-technical-requirements-registration-pharmaceuticals-human-use-topic-e-2-clinical-safety-data-management-definitions-and-standards-expedited-reporting-step_en.pdf.

[25] Bates D.W., Miller E.B., Cullen D.J., Burdick L., Williams L., Laird N., Petersen L.A., Small S.D., Sweitzer B.J., ADE Prevention Study Group (1999). Patient risk factors for adverse drug events in hospitalized patients. Arch. Intern. Med..

[26] Bertsche T., Pfaff J., Schiller P., Kaltschmidt J., Pruszydlo M.G., Stremmel W., Walter-Sack I., Haefeli W.E., Encke J. (2010). Prevention of adverse drug reactions in intensive care patients by personal intervention based on an electronic clinical decision support system. Intensive Care Med..

[27] Meyboom R.H.B., Royer R.J. (1992). Causality classification at pharmacovigilance centres in the european community. Pharmacoepidem. Drug Safe.

[28] Rottenkolber D., Schmiedl S., Rottenkolber M., Farker K., Saljé K., Mueller S., Hippius M., Thuermann P.A., Hasford J. (2011). Adverse drug reactions in Germany: Direct costs of internal medicine hospitalizations. Pharmacoepidemiol. Drug Saf..

[29] Nemec M., Koller M.T., Nickel C.H., Maile S., Winterhalder C., Karrer C., Laifer G., Bingisser R. (2010). Patients presenting to the emergency department with non-specific complaints: The Basel Non-specific Complaints (BANC) study. Acad. Emerg. Med..

[30] Otto R., Roller R.E., Iglseder B., Dovjak P., Lechleitner M., Sommeregger U., Benvenuti-Falger U., Böhmdorfer B., Gosch M. (2010). Komplikationen durch Diuretikatherapie bei geriatrischen Patienten. Wien. Med. Wochenschr..

[31] De Jong M.R., Van der Elst M., Hartholt K.A. (2013). Drug-related falls in older patients: Implicated drugs, consequences, and possible prevention strategies. Ther. Adv. Drug Saf..

[32] Gross K., King A.N., Steadman E. (2021). Impact of a Pharmacy-Led Fall Prevention Program for Institutionalized Older People. Sr. Care Pharm..

